# Publisher Correction: Mental navigation in the primate entorhinal cortex

**DOI:** 10.1038/s41586-024-07737-x

**Published:** 2024-07-04

**Authors:** Sujaya Neupane, Ila Fiete, Mehrdad Jazayeri

**Affiliations:** 1 McGovern Institute for Brain Research, Massachusetts Institute of Technology, Cambridge, MA USA; 2 Department of Brain and Cognitive Sciences, Massachusetts Institute of Technology, Cambridge, MA USA

**Keywords:** Cognitive neuroscience, Computational neuroscience

Correction to: *Nature* 10.1038/s41586-024-07557-z Published online 12 June 2024

This article was originally published under standard Springer Nature license (© The Author(s), under exclusive licence to Springer Nature Limited). It is now available as an open-access paper under a Creative Commons Attribution 4.0 International license, © The Author(s). Also, there were errors in the Fig. 3b *y*-axis labels where, in the text now reading “Cross-validated *R*^2^ at offset”, “offset” originally appeared as “onset”. The errors have been corrected in the HTML and PDF versions of the article.

