# Supplementary material for: Mental navigation in the primate entorhinal cortex
Source: Nature. 2024 Jun 12;630(8017):704–11. doi: 10.1038/s41586-024-07557-z (PMC11224022; doi:10.1038/s41586-024-07557-z)
Supplement: Supplementary file 1 — This file contains Supplementary Discussion and Supplementary References. [file 41586_2024_7557_MOESM1_ESM.docx]

**Vector production via mental navigation in the entorhinal cortex**

Sujaya Neupane, Ila Fiete and Mehrdad Jazayeri

**Supplementary information**

**1. Supplementary Discussion**

**The identity of periodic neurons in primate EC**

The first interpretation of the periodic neurons is that they could be homologous to landmark cells recorded in rodents during navigation. Previous work suggests that landmark neurons are modulated during the presence of visible landmarks and not when landmarks are invisible^28^. Accordingly, when we applied our analysis to landmark neurons in a previous study^28^, cross-correlations were preserved across epochs with visible landmarks but abolished when the landmarks were invisible (Fig S7; H0: correlation for region B visible = correlation for region B invisible; one-tailed 2 sample t-test(198)=13.73, p<<0.0001). Furthermore, the preserved correlations during the inference epoch in the MNAV task make it unlikely that these neurons were landmark cells. During this epoch, the animals were presented with the start and target landmarks. If these neurons were landmark cells, we would expect the strong visual inputs from the visible start and target landmarks to weaken correlations. However, we found no such drop in cross-correlations (Fig S5b,c; r = 0.93, p<<.0001 for monkey A and r = .83, p<<.0001, for monkey M) without a significant drop in magnitude compared to the navigation epoch (right-tailed 2 sample t-test2(418)=1.4928, p=0.068 for monkey A and t-test2(592)= -9.1, p=1 for monkey M, Figure S5b,c). Together, these results are inconsistent with the interpretation that our task-modulated neurons are canonical landmark cells. However, we know nearly nothing about landmark cells in the primate brain. It is therefore possible that the EC neurons we have identified function as landmark cells with the additional capacity to express attractor dynamics. The second interpretation is that our periodic task-modulated neurons may be homologous to GC cells in rodents. The GC interpretation is supported by the presence of endogenous periodicity and the preserved cell-cell correlations but is questionable given the significantly tighter clustering of the relative phase distribution of cell-cell cross-correlation in our data. This clustering may be partially due to the plasticity of connections to putative landmark cells but the clustering was stronger in our data compared to the GC model units (Fig S7e-g). Moreover, many studies have found that the firing patterns of GCs can remap rapidly in response to both environmental factors^65–67^ and internal variables^68^ without the need for slow synaptic modifications. Yet, other studies have found evidence for slow learning within the grid system^18,69^, as predicted by our model. Therefore, at this stage, we cannot make a definitive statement about whether GCs undergo slow synaptic modifications during mental navigation. One experimental modification that could shed light on this question is to record from this cell population in a variant of our task with irregular spacing between landmarks. In that scenario, we would be able to identify these neurons as GC more definitively if they maintain their periodicity. Therefore, we conclude that either the periodic neurons in our dataset are not homologous to GC or that the attractor network supporting grid-like activity in primates differs from rodents. A final alternative is that the periodic neurons in our population are neither landmark cells nor GCs but are part of an attractor network within the functional architecture of the entorhinal cortex that is capable of producing memory traces for the landmarks^22–24^. Large-scale recording with tight behaviorally controlled experiments will help arbitrate between the three alternative identities of our periodic neurons.

**4. Supplementary Videos**

**Supplementary video 1.** **Navigation to Sample (NTS) task.** Reconstructed example trials of NTS with randomly sampled start and target landmark images.

**Supplementary video 2.** **Mental navigation (MNAV) task.** Reconstructed example trials of MNAV with randomly sampled start and target landmark images.

**5. Supplementary References**

65. Barry, C., Hayman, R., Burgess, N. & Jeffery, K. J. Experience-dependent rescaling of entorhinal grids. *Nat. Neurosci.* **10**, 682–684 (2007).

66. Fyhn, M., Hafting, T., Treves, A., Moser, M.-B. & Moser, E. I. Hippocampal remapping and grid realignment in entorhinal cortex. *Nature* **446**, 190–194 (2007).

67. Krupic, J., Bauza, M., Burton, S., Barry, C. & O’Keefe, J. Grid cell symmetry is shaped by environmental geometry. *Nature* **518**, 232–235 (2015).

68. Low, I. I. C., Williams, A. H., Campbell, M. G., Linderman, S. W. & Giocomo, L. M. Dynamic and reversible remapping of network representations in an unchanging environment. *Neuron* **110**, 903 (2022).

69. Boccara, C. N., Nardin, M., Stella, F., O’Neill, J. & Csicsvari, J. The entorhinal cognitive map is attracted to goals. *Science* **363**, 1443–1447 (2019)
