# Supplementary material for: Mental navigation in the primate entorhinal cortex
Source: Nature. 2024 Jun 12;630(8017):704–11. doi: 10.1038/s41586-024-07557-z (PMC11224022; doi:10.1038/s41586-024-07557-z)
Supplement: Supplementary file 2 — Reporting Summary [file 41586_2024_7557_MOESM2_ESM.pdf]

nature portfolio

Corresponding author(s): Mehrdad Jazayeri

Last updated by author(s): Mar 21, 2024

Reporting Summary

Nature Portfolio wishes to improve the reproducibility of the work that we publish. This form provides structure for consistency and transparency in reporting. For further information on Nature Portfolio policies, see our Editorial Policies and the Editorial Policy Checklist.

Statistics

For all statistical analyses, confirm that the following items are present in the figure legend, table legend, main text, or Methods section.

n/a	Confirmed	
☐	☒	The exact sample size (*n*) for each experimental group/condition, given as a discrete number and unit of measurement
☐	☒	A statement on whether measurements were taken from distinct samples or whether the same sample was measured repeatedly
☐	☒	The statistical test(s) used AND whether they are one- or two-sided<br>*Only common tests should be described solely by name; describe more complex techniques in the Methods section.*
☐	☒	A description of all covariates tested
☐	☒	A description of any assumptions or corrections, such as tests of normality and adjustment for multiple comparisons
☐	☒	A full description of the statistical parameters including central tendency (e.g. means) or other basic estimates (e.g. regression coefficient) AND variation (e.g. standard deviation) or associated estimates of uncertainty (e.g. confidence intervals)
☐	☒	For null hypothesis testing, the test statistic (e.g. *F*, *t*, *r*) with confidence intervals, effect sizes, degrees of freedom and *P* value noted<br>*Give P values as exact values whenever suitable.*
☐	☒	For Bayesian analysis, information on the choice of priors and Markov chain Monte Carlo settings
☒	☐	For hierarchical and complex designs, identification of the appropriate level for tests and full reporting of outcomes
☒	☐	Estimates of effect sizes (e.g. Cohen's *d*, Pearson's *r*), indicating how they were calculated

Our web collection on statistics for biologists contains articles on many of the points above.

Software and code

Policy information about availability of computer code

Data collection	Behavioral console was controlled via MWorks (mworks.github.io, version 0.9). Eye movements were collected using Eyelink 1000 (SR Research Ltd, Ontario, Canada). Joystick/Hand movements were collected using (PCIe6251 board, National Instruments, TX). 32- and 64-channel laminar probes (V-probe, Plexon Inc., TX) were used for extracellular recordings. A motorized micro-manipulator was used for targeting the region of interest (Narasighe Inc.). Recorded signals were preprocessed using OpenEphys (OpenEphys Inc., Lisbon, Portugal). Kilosort 2.0 was used for spike sorting. A python-based GUI (phy) was used to verify spike sorting quality manually.
Data analysis	Data analysis was performed using Matlab.

For manuscripts utilizing custom algorithms or software that are central to the research but not yet described in published literature, software must be made available to editors and reviewers. We strongly encourage code deposition in a community repository (e.g. GitHub). See the Nature Portfolio guidelines for submitting code & software for further information.

Data

Policy information about availability of data

All manuscripts must include a data availability statement. This statement should provide the following information, where applicable:

- Accession codes, unique identifiers, or web links for publicly available datasets
- A description of any restrictions on data availability
- For clinical datasets or third party data, please ensure that the statement adheres to our policy

The data used to generate the associated figures and the URL for access will be made available on a public repository.

Field-specific reporting

Please select the one below that is the best fit for your research. If you are not sure, read the appropriate sections before making your selection.

☒ Life sciences ☐ Behavioural & social sciences ☐ Ecological, evolutionary & environmental sciences

For a reference copy of the document with all sections, see nature.com/documents/nr-reporting-summary-flat.pdf

Life sciences study design

All studies must disclose on these points even when the disclosure is negative.

Sample size	We used n=2 monkeys
Data exclusions	We did not exclude any data relevant to this study. We have included all cases when specific aspects of the data was used in a figure or analysis.
Replication	All results reported were replicated by cross-validation across different portions of the data. In all cases where the data was pooled across conditions, the analysis was also performed on individual conditions to ensure the results were robust.
Randomization	All experimental conditions (trials and trial types) were presented randomly.
Blinding	The trial conditions were randomly interleaved and therefore both the experimenters and the subjects were blind to the experimental conditions/groupings.

Reporting for specific materials, systems and methods

We require information from authors about some types of materials, experimental systems and methods used in many studies. Here, indicate whether each material, system or method listed is relevant to your study. If you are not sure if a list item applies to your research, read the appropriate section before selecting a response.

Materials & experimental systems

n/a	Involved in the study	
☒	☐	Antibodies
☒	☐	Eukaryotic cell lines
☒	☐	Palaeontology and archaeology
☐	☒	Animals and other organisms
☒	☐	Human research participants
☒	☐	Clinical data
☒	☐	Dual use research of concern

Methods

n/a	Involved in the study	
☒	☐	ChIP-seq
☒	☐	Flow cytometry
☒	☐	MRI-based neuroimaging

Animals and other organisms

Policy information about studies involving animals; ARRIVE guidelines recommended for reporting animal research

Laboratory animals	2 rhesus macaque monkeys (Macaca mulatta).
Wild animals	Study did not involve wild animals.
Field-collected samples	Study did not involve field-collected samples.
Ethics oversight	All procedures were performed in compliance with the guideline of National Institutes of Health and the American Physiological Society, and approved by the MIT Committee on Animal Care.

Note that full information on the approval of the study protocol must also be provided in the manuscript.
