# Supplementary material for: Mental navigation in the primate entorhinal cortex
Source: Nature. 2024 Jun 12;630(8017):704–11. doi: 10.1038/s41586-024-07557-z (PMC11224022; doi:10.1038/s41586-024-07557-z)
Supplement: Supplementary file 3 — Supplementary Tables [file 41586_2024_7557_MOESM3_ESM.pdf]

	Property	Left only	Right only	Both directions	Total
Monkey A	Periodicity	90 (39%)	85(37%)	56 (24%)	231/614
	Ramping	27 (46%)	23 (40%)	8 (14%)	58/614
Monkey M	Periodicity	142 (46%)	101(32%)	68 (22%)	311/864
	Ramping	40 (39%)	28 (27%)	33 (33%)	101/864

Table 1: Cell count for periodic and ramping neurons grouped by direction of movement.

	Downward ramp	Upward ramp	Ramping only	Periodic only	Both
Right, Monkey A	16	15	29	139	2
Left, Monkey A	32	3	31	142	4
Right, Monkey M	4	57	46	154	15
Left, Monkey M	14	59	58	195	15

Table 2: Cell count for task-modulated neurons broken into subgroups of upward ramping, downward ramping, ramping only, periodicity only, and both ramping and periodicity, for the two directions.

Electrodes from Plexon Inc.	Length (mm)	Diameter (um)	Electrode spacing (um)	Recording span (mm)
32-channel V-probe	100	240	100	3
64-channel V-probe	120	360	50	3

Table 3: Technical specifications of the recording electrodes.
